# Achieving Millennium Development Goals 4 and 5: Do every mother and child really count?

**DOI:** 10.3325/cmj.2013.54.107

**Published:** 2013-04

**Authors:** Ivana Kolčić

**Affiliations:** Croatian Centre for Global Health, Faculty of Medicine, University of Split, Split, Croatia

Is there any human experience that could be compared to the kaleidoscope of emotions and thoughts rushing through the soul of every mother just after the birth of her child? What measures would parents be willing to undertake if their child was seriously ill? How could they find consolation if their child died from preventable or easily treatable causes, such as pneumonia or diarrhea? Although I write this from the position of a mother from a developed country, it does not mean that mothers from low- and middle-income countries (LMIC) feel any differently when facing such a threat.

At the dawn of the new millennium, the burden of disease and death estimates in developing countries was disappointingly – if not scandalously large. The same is valid for poverty and poor economic perspective in these countries, which today are home to more than two thirds of humankind. For instance, the mortality of children younger than five years in 1990 was estimated to 11.9 million (1). In 1990-92, as many as 1.8 billion people in the developing countries were living with less than US $1.25 a day (2) and 824 million people were hungry (3). Even if leaving other important issues aside, such as access to water and sanitation, economic inequity and gender inequality, political instability and widespread corruption, human rights, and weak health systems, these simple indicators were a signal enough for urgent and imperative actions. United Nations took the lead in the preparation and implementation of the actions that were needed (4).

Since the adoption of the United Nation's Millennium Declaration in 2000, the foundations for better safeguard of humanity have been laid through the formulation and acceptance of the Millennium Development Goals (MDG). These Goals serve for advocacy and guidance in united efforts to reach better health, better lives, and better societies world-wide. Basically, MDGs are conceived with the aim of alleviating at least some of the suffering that the majority of people still experience globally and to ensure basic human rights for all humanity (4).

Who can contribute to the MDGs? The answer is simple: everyone can! Today there are hundreds of international agencies, foundations, and non-governmental organizations capable of bringing the aid to LMIC. Some of the UN partners on MDGs are United Nations Development Programme, World Bank, UN Children’s Fund, the World Health Organization, International Monetary Fund, Food and Agriculture Organization, International Labour Organization, Bill and Melinda Gates Foundation, and many more.

If one considered each of the eight MDGs on their own in the year 2000, when they were proposed, they would have all seemed hard, or even impossible, to reach. However, these goals are very intertwined. For instance, if universal primary education for all was achieved (MDG 2), this would also improve maternal health (MDG 5), and the way in which mothers care for their children, recognize danger signs, and seek care. This, in turn, would contribute to improving child health and reducing child mortality rates (MDG 4). Education would further reduce poverty and hunger (MDG 1), by empowering the population, which would reduce undernutrition-related risks among mothers and children (5). Education helps in combating HIV/AIDS (MDG 6) and other infectious and non-communicable diseases, ensures the capacity for achieving environmental sustainability (MDG 7), and provides building blocks for global partnership development (MDG 8) and gender equality (MDG 3). Finally, if we succeed in alleviating the most resistant underlying scourge of them all – widespread poverty – this would provide better grounds for success in reaching of all the other MDGs (6).

United Nations' report on MDGs in 2012 stated: “The Millennium Development Goals (MDGs) agreed to by world leaders over a decade ago have achieved important results. Working together, Governments, the United Nations family, the private sector and civil society have succeeded in saving many lives and improving conditions for many more. The world has met some important targets — ahead of the deadline” (4). The MDG that has been met ahead of plan is the MDG 1 – to reduce the global poverty rate by half. Estimates indicate that the rate of people living on less than US $1.25 a day fell from 47% in 1990 to 24% in 2010 (4). In contrast to these figures, 850 million people in the world in 2006-2008 still lived in hunger, indicating almost no progress from 1990s, especially in sub-Saharan Africa. Child undernutrition has hardly been improved, probably reflecting the food price crisis and global financial crisis (4,5).

MDG 4, which requires a two-thirds reduction in under-five mortality rate (U5MR) between 1990 and 2015, succeeded only partially, with a reduction of 4.2 million deaths world-wide: from about 12 million deaths in 1990 to under 8 million deaths in 2010 (1). The promising momentum is the acceleration in progress in LMICs, especially in sub-Saharan Africa, a region with world's highest child mortality rate, where the average rate of U5MR reduction has doubled, from 1.2% a year during 1990-2000 to 2.4% during 2000-2010 (4). Despite these positive changes and under-five mortality rate decline of more than one third, the progress is still too slow to reach the target by 2015 (4). To prevent us from failing, urgent actions and additional measures are needed to surpass the previous success, especially in combating the neonatal mortality, which accounts for the highest proportion of U5MR. Maternal mortality goal (MDG 5) called for a reduction by three quarters between 1990 and 2015. Important improvements were achieved, but the overall progress is still slow (7). Without substantial acceleration this goal will not be met by 2015 (4).

Tracking the progress of reaching MDGs is pivotal for maintaining the drive to achieve these goals in each particular country, region, and the world. Most of the reports and analyses of MDGs tracking in recent years have been published by a cluster of medical journals particularly focused on global health issues. Those journals should also be congratulated for their substantial role in facilitating the progress, and they include (but are not limited to) *the Lancet*, *PLOS* (Public Library of Science) group of journals, *The BMC-series* (BioMed Central) journals, *Bulletin of the World Health Organization*, and others. Nine out of ten most cited papers dealing with MDGs were published in *the Lancet* (8). Its “Child Survival” series has been pivotal in raising awareness that the UN's MDG 4 cannot be achieved without an increased focus on preventing and treating childhood infections, particularly pneumonia and diarrhea in low- and middle-income countries (9). Another pivotal publication regarding the MDG 4 and MDG 5 is the World Health Report 2005, where the former Director-General Dr Lee Jong-Wook stated: *“Mothers, the newborn and children represent the well-being of a society and its potential for the future. Their health needs cannot be left unmet without harming the whole of society.”* (10). The question that arises is: “Are we willing to risk the harm to the whole society by not taking into account every mother and every child?”

What does it really take to make *every* mother and *every* child count, not only in mortality statistics, but also in ensuring their better lives and improved quality of living conditions? It takes proper nourishment, vaccination, access to health care, early and accurate diagnosis, and finally, appropriate treatment. All of these segments are attainable if education and economic stability are ensured. We should not forget that the year 2015 is just around the corner, and approaching rather fast.

This issue of the *Croatian Medical Journal* presents five articles dealing with MDG 4 and MDG 5. All the articles are a result of collaboration of Croatian researchers interested in global health topics and their international counterparts. In a logical sequence they cover a continuum of maternal, newborn, and child health topics. Chen et al and UNICEF's China office compare the quality of antenatal care in public and private sector in rural China (11), while Wu et al investigate approaches to improve the intake of nutritious foods in infants and young children (12). Jackson et al offer a series of meta-analyses to summarize the importance and contribution of different risk factors to severe episodes of acute lower respiratory infections (ALRI) (13). Lukšić et al add to this work by exploring the viral causes of ALRI (14) and assessing the effectiveness of influenza vaccination (15).

## References

[1] Liu L, Johnson HL, Cousens S, Perin J, Scott S, Lawn JE (2012). Global, regional, and national causes of child mortality: an updated systematic analysis for 2010 with time trends since 2000.. Lancet.

[2] Department of Economic and Social Affairs. Rethinking poverty. Report on the world social situation 2010. New York (NY): United Nations; 2009.

[3] Food and Agriculture Organization. Food deprivation trends and progress: Hunger reduction targets in the developing world. FAO, 2006. Available from: http://www.fao.org/fileadmin/templates/ess/documents/food_security_statistics/working_paper_series/WP002e.pdf*.* Accessed: April 24, 2013.

[4] Millennium Development Goals Report 2012. United Nations, New York, 2012. Available from: http://www.un.org/millenniumgoals/pdf/MDG%20Report%202012.pdf*.* Accessed: April 24, 2013.

[5] Kolčić I (2012). Double burden of malnutrition: A silent driver of double burden of disease in low- and middle-income countries.. J Glob Health..

[6] Chopra M, Campbell H, Rudan I (2012). Understanding the determinants of the complex interplay between cost-effectiveness and equitable impact in maternal and child mortality reduction.. J Glob Health..

[7] Nair H, Panda R (2011). Quality of maternal healthcare in India: Has the National Rural Health Mission made a difference?. J Glob Health..

[8] Reuters T. Web of Science. Available from: http://apps.webofknowledge.com/CitationReport.do?product=UA&search_mode=CitationReport&SID=X18dNgGHaHhM2bcjpbl&page=1&cr_pqid=8&viewType=summary Accessed: February 23, 2013.

[9] Rudan I, El Arifeen S, Black RE, Campbell H (2007). Childhood pneumonia and diarrhoea: setting our priorities right.. Lancet Infect Dis.

[10] World Health Organization. World Health Report 2005: make every mother and child count. Geneva (Switzerland): WHO; 2005.

[11] Chen L Dai Y Zhang Y Wu Q Rudan D Saftic V A comparison between antenatal care quality in public and private sector in rural Hebei, China. Croat Med J 2013 54 146 56doi: 10.3325/cmj.2013.54.146 23630142 10.3325/cmj.2013.54.146 PMC3641873

[12] Wu Q van Velthoven MHMMT Chen L Car J Rudan D Saftic V Improving the intake of nutritious food in children aged 6-23 months in Wuyi County, China: a multi-method approach. Croat Med J 2013 54 157 70 doi: 10.3325/cmj.2013.54.157 23630143 10.3325/cmj.2013.54.157 PMC3662389

[13] Jackson S Mathews KH Pulanic D Rudan I Campbell H Nair H Risk factors for severe acute lower respiratory infections in children: a systematic review and meta-analysis. Croat Med J 2013 54 110 21doi: 10.3325/cmj.2013.54.110 23630139 10.3325/cmj.2013.54.110 PMC3641871

[14] Luksic I Kearns PK Scott F Rudan I Campbell H Nair H Viral etiology of hospitalized acute lower respiratory infections in children under 5 years of age: a systematic review and meta-analysis. Croat Med J 2013 54 122 34 doi: 10.3325/cmj.2013.54.122 23630140 10.3325/cmj.2013.54.122 PMC3641872

[15] Luksic I Clay S Pulanic D Rudan I Campbell H Nair H Effectiveness of seasonal influenza vaccines in children: a systematic review and meta-analysis. Croat Med J 2013 54 135 45doi: 10.3325/cmj.2013.54.135 23630141 10.3325/cmj.2013.54.135 PMC3662362

